# Industry Speed Bumps on Local Tobacco Control in Japan? The Case of Hyogo

**DOI:** 10.2188/jea.JE20150001

**Published:** 2015-07-05

**Authors:** Keiko Yamada, Nagisa Mori, Mina Kashiwabara, Sakiko Yasuda, Rumi Horie, Hiroshi Yamato, Loic Garçon, Francisco Armada

**Affiliations:** 1 World Health Organization, Centre for Health Development, Kobe, Japan; 1 世界保健機関（WHO）構健康開発総合研究センター（WHO神戸センター）; 2 Public Health, Department of Social Medicine, Graduate School of Medicine, Osaka University, Suita, Osaka, Japan; 2 大阪大学大学院 医学系研究科 社会医学講座 公衆衛生学; 3 Health & Welfare Department, Hyogo Prefectural Government, Kobe, Japan; 3 兵庫県 健康福祉部 健康局 医務課; 4 Department of Social and Preventive Epidemiology, Graduate School of Medicine, The University of Tokyo, Tokyo, Japan; 4 東京大学大学院 医学系研究科 社会予防疫学分野; 5 World Health Organization, Western Pacific Regional Office, Manila, Philippines; 5 世界保健機関（WHO）西太平洋地域事務局; 6 Department of Global Health and Socio-epidemiology, School of Public Health, Kyoto University, Kyoto, Japan; 6 京都大学 大学院医学研究科 社会健康医学系専攻 国際保健学講座 社会疫学分野; 7 Tokyo Development Learning Center, the World Bank, Tokyo, Japan; 7 世界銀行東京開発ラーニングセンター; 8 Department of Health Development, Institute of Industrial Ecological Sciences, University of Occupational and Environmental Health, Kitakyushu, Fukuoka, Japan; 8 産業医科大学 産業生態科学研究所 健康開発科学研究室

**Keywords:** smoking, tobacco smoke pollution, legislation, local government, Japan

## Abstract

**Background:**

Despite being a signatory since 2004, Japan has not yet fully implemented Article 8 of the World Health Organization’s Framework Convention on Tobacco Control regarding 100% protection against exposure to second-hand smoke (SHS). The Japanese government still recognizes designated smoking rooms (DSRs) in public space as a valid control measure. Furthermore, subnational initiatives for tobacco control in Japan are of limited effectiveness. Through an analysis of the Hyogo initiative in 2012, we identified key barriers to the achievement of a smoke-free environment.

**Methods:**

Using a descriptive case-study approach, we analyzed the smoke-free policy development process. The information was obtained from meeting minutes and other gray literature, such as public records, well as key informant interviews.

**Results:**

Hyogo Prefecture established a committee to propose measures against SHS, and most committee members agreed with establishing completely smoke-free environments. However, the hospitality sector representatives opposed regulation, and tobacco companies were allowed to make a presentation to the committee. Further, political power shifted against completely smoke-free environments in the context of upcoming local elections, which was an obvious barrier to effective regulation. Throughout the approving process, advocacy by civil society for stronger regulation was weak. Eventually, the ordinance approved by the Prefectural Assembly was even weaker than the committee proposal and included wide exemptions.

**Conclusions:**

The analysis of Hyogo’s SHS control initiative shed light on three factors that present challenges to implementing tobacco control regulations in Japan, from which other countries can also draw lessons: incomplete national legislation, the weakness of advocacy by the civil society, and the interference of the tobacco industry.

## 抄録

**背景:**

日本は「たばこの規制に関する世界保健機関枠組条約」を2004年に批准したが、その第8条である公共の場所における「たばこの煙にさらされることからの保護」を未だ完全に実施できていない。そればかりか、日本政府は公共の場所における喫煙室の設置を妥当な方策であると許容している。さらに、地方自治体によるたばこ規制も日本においては限定的である。本研究は2012年の兵庫県によるたばこ規制の取り組み（受動喫煙防止条例の制定）を分析し、公共の場における禁煙環境の達成において何が障壁となっているのかを明らかにした。

**方法:**

記述的ケーススタディの手法を用い、受動喫煙防止条例の成立過程を分析した。データは議事録や、公的記録、並びに施策関係者に対するインタビューを用いた。

**結果:**

兵庫県は受動喫煙防止の条例化を提議するための委員会を設立し、ほとんどの委員会メンバーは飲食店等を含む公共的な場所（屋内）の完全な禁煙化に賛成した。しかし、委員のうち飲食業と宿泊業の代表者は条例による禁煙に反対した。加えて、委員会会議でたばこ会社に発言の機会が設けられた。さらに、条例化の最中に開催された地方選挙によって県議会の政権が交代し、このことも公共的な場所の完全な禁煙を定めた条例の制定に対して影響した。また、条例制定の過程を通して、市民団体による公共的な場所（屋内）の完全禁煙化を求める働きかけも他国と比して不活発であった。最終的に県議会が制定した条例は、委員会の提案内容より規制が弱い、広範囲の例外規定を含んだものとなった。

**結論:**

兵庫県の取り組みを分析することで、三つの要素（不完全な国の法規制、市民団体による活動の弱さ、たばこ産業による干渉が明らかになった。これらは他国における今後の教訓となり、我が国のたばこ規制における重要な課題ともいえる。

## INTRODUCTION

Article 8 of the World Health Organization (WHO) Framework Convention on Tobacco Control (FCTC) requests its parties to implement a 100% smoke-free policy. Despite being a signatory since 2004, Japan has not yet fully implemented its provisions.^1^^,^^2^

In 2000, the Ministry of Health, Labour and Welfare (MHLW) of Japan initiated a national health promotion movement (known as *Health Japan 21*), which set tobacco-related indicators focusing on the dissemination of evidenced-based information on the health impact of smoking.^3^ Adoption of the *Health Promotion Act* in 2002 provided a legal basis for protecting people from second-hand smoke (SHS). However, smoking in many public places remains unrestricted.^4^

Smoke-free legislation passed at the national level is ideal for providing nationwide coverage. In its absence, many subnational governments worldwide have adopted local smoke-free policies. Subnational smoke-free intervention, which may complement or precede national law, is becoming a popular option for increasing numbers of local governments.

While there is no national smoke-free law in Japan, municipalities have implemented street smoking bans. By the end of 2009, more than 100 municipalities had banned smoking on streets. However, these bans provide limited protection from SHS only in specific outdoor zones.^5^^,^^6^

An important consideration in understanding tobacco control in Japan is that the Ministry of Finance has been the major shareholder in Japan Tobacco Inc. (JT), Japan’s largest tobacco corporation. Some scholars have highlighted this connection as a hindrance to tobacco control.^7^^–^^10^

In 2009, Kanagawa became the first prefecture in Japan to pass an ordinance to restrict smoking in indoor public places.^11^ In 2010, Hyogo Prefecture established a consultative committee as a preliminary step to develop policy on smoking in public places and adopted a similar ordinance.^12^

The objectives of this study were to describe and analyze the legislative process for protecting people from SHS. Through the analysis, we identified key elements that either facilitate or interfere with developing national and subnational smoke-free laws in Japan.

## METHODS

The study, which employed a descriptive case study approach,^13^ was conducted between November 2011 and February 2015. Information on the process between January 2010 and September 2012, especially on the discussions of the consultative committee, was collected through a review of gray literature (eg, meeting minutes and public records). Internet searches to obtain online news articles were made using the terms “Hyogo Prefecture ordinance on the prevention of SHS”, “Hyogo Prefecture”, and similar terms in Japanese. The decision to carry out the research was taken after the approval of the ordinance, so notes taken during the process did not follow a research protocol. Key informant interviews were conducted with five stakeholders, including members of the committee and staff of the Hyogo Prefectural SHS Prevention Office. The information was analyzed to shed light on key factors that influenced the policy development. As a guide to the analysis, we compared the adopted ordinance with WHO’s *Model ordinance*^14^ and the development process with the recommendations in the WHO’s *Twelve steps to a smoke-free city*.^14^

No interviews were conducted with local parliament members, limiting the analysis of that part of the process. Generalization to other cities within and outside Japan would be limited by the specific circumstantial and contextual conditions of Hyogo during the time of the studied initiative. Another limitation to consider, often related to case-studies, is the potential bias from the researchers due to their own subjectivity or from others involved in the process,^15^ although this has been disregarded by Flyvberg.^16^

## RESULTS

### Overview and background

In 2000, Hyogo Prefecture launched an anti-SHS initiative, including a policy for *smoking separation* (separating smoking and non-smoking areas) in all public places and workplaces. In 2004, Hyogo Prefecture adopted the *Hyogo SHS prevention guidelines* aiming at the implementation of an indoor smoking ban by March 2011.^17^ The guideline called for a complete smoking ban in public spaces, as well as preventing SHS exposure at home when pregnant women or infants are present.^17^ However, a survey conducted in 2008 by Hyogo Prefecture revealed that only 58.5% of government buildings were smoke-free.^18^

In February 2010, MHLW addressed an official letter to governors and mayors encouraging the implementation of policies to prevent SHS.^19^ Referring to WHO FCTC Article 8, the letter noted the necessity of implementing 100% smoke-free environments in public places, including outdoor spaces frequented by children and women. However, it recognized *smoking separation* as a valid measure where *directors or managers of the establishments find it difficult to ban smoking completely* and stated that the Japan Finance Corporation, under the Ministry of Finance, had made funds available for introducing smoking separations.^19^ In 2011, the funder was changed from the Japan Finance Corporation to MHLW.^20^

These two policy documents—*the Hyogo SHS prevention guideline* and the letter from the MHLW—were used by the Governor of Hyogo to form the *Hyogo Consultation Committee for SHS Prevention Measures* in 2010.^21^ The Director of Hyogo Prefectural Hospital was elected as its chairperson.

Through committee meetings, the proposed content for the ordinance was prepared. The outline of the ordinance received public comments before the first draft of the ordinance was submitted to the Prefectural Assembly by the Department of Health and Welfare (DoHW) (Figure).

**Figure.**
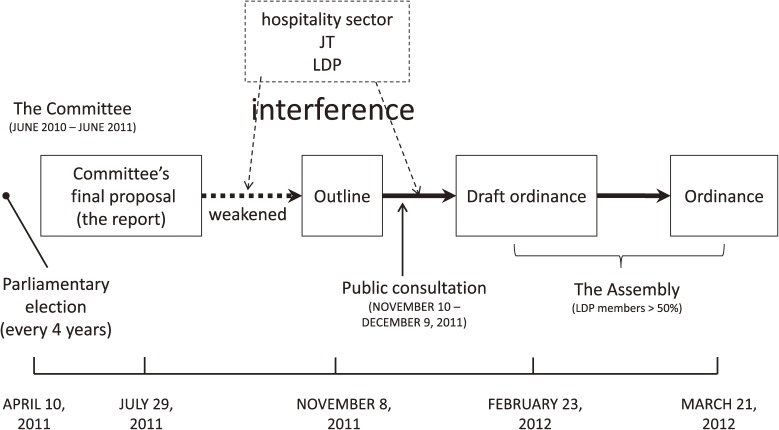
The process of establishing the local ordinance for protects people from second-hand smoke in Hyogo Prefecture. JT, Japan Tobacco Inc.; LDP, Liberal Democratic Party.

### Committee meetings (June 2010 to June 2011)

The committee comprised 15 members representing local government, academia, civil society, the media, health-care providers, and the hospitality industry in and outside Hyogo Prefecture. A staff member from a WHO office located in Hyogo Prefecture was also included.^22^ The Governor, the chairperson, and the DoHW selected the members.

The committee held a total of nine meetings (though originally only four were planned). The DoHW and the chairperson drew up the agenda. Each meeting had seats for about 10 observers on a first-come, first-served basis; these were mostly taken by tobacco industry representatives.

At the first meeting (June 2, 2010), the chairperson explained the role of the committee to propose specific measures against SHS exposure in Hyogo Prefecture. The main discussion was on whether or not to introduce a prefectural ordinance. All committee members, except for the representatives of restaurant and hotel associations, supported introducing local ordinance.

The second meeting (July 14, 2010) was mainly devoted to the discussion of contrasting views presented by invited speakers: a representative of the tobacco industry and a local tobacco control advocate. Allegedly on grounds of fairness, the tobacco industry (Philip Morris [PM] and JT) were invited to express their views. JT introduced examples from other countries where the hospitality industry claimed to experience a negative economic impact due to smoking bans.^19^ A representative of a local civil society organization, Hyogo Tobacco Free Advocacy (HTFA), stressed Japan’s needed compliance with the WHO FCTC and that Hyogo’s ordinance had to ensure completely smoke-free environments. The results of an online survey completed by 2289 Hyogo Prefecture residents in April 2010 showed high support for regulating smoking (nearly 80%), even in hospitality premises (over 60%).

At the third (August 6, 2010) and fourth (September 10, 2010) meetings, the DoHW shared the results of a survey targeting customers in hospitality venues, revealing high support (nearly 70%) for regulating smoking in public places. Most members agreed that Hyogo Prefecture must enforce smoke-free environments to protect public health.

At the fifth meeting (September 21, 2010), the committee seemed to agree to include in the proposed ordinance a separation of smoking and non-smoking areas in private business facilities. However, committee members noted the need to clarify to which kinds of establishment this separation measure would apply, and that it should be considered a temporary measure until a 100% smoking ban is enforced.

The sixth meeting (October 18, 2010) addressed provisions (including the transitional period and exceptions) for private business facilities, and it was reaffirmed that *separation of smoking areas* was not adequate. This discussion continued during the seventh meeting (December 6, 2010) with a focus on designated smoking rooms (DSRs). Some members felt that it was unacceptable to use the term “DSRs” in the ordinance as it would jeopardize a future 100% smoking ban.

The committee entered its final stage. The draft report prepared for the Governor was shared for feedback at the eighth meeting on May 23, 2011 and approved in the final meeting on June 30, 2011. However, the members from the hospitality industry submitted a statement opposing the draft report and a proposed ordinance. The DoHW expressed its concern that “the freedom of economic activities” would be unfairly affected by a complete smoking ban.^19^ The DoHW further revised the report, and its final version was officially released to the public on July 29, 2011.

While the committee meetings were in session, an important political event took place: the quadrennial Hyogo Prefectural Assembly election was held on April 10, 2011. Liberal Democratic Party (LDP) gained the majority of seats.^23^ On September 2, 2011, a meeting between Hyogo prefectural representatives of the LDP and the hospitality industry was held where the latter strongly opposed the ordinance. On December 21, 2011, the LDP submitted a request at the Hyogo Prefectural Assembly to weaken the regulation for economic reasons (Figure).^26^

According to the outline of the ordinance officially announced on November 8, 2011, all public places should be smoke-free (Figure). Exceptionally, *for the time being*, smoking facilities for the hospitality industry were allowed. In hospitality establishments of over 75 m^2^, either more than 1/3 of the area should be consistently non-smoking, or “non-smoking hours” should be set, during which smoking is completely banned.

Subsequently, Hyogo Prefecture opened a public consultation, making the content of the ordinance accessible online and accepting feedback by e-mail, fax, or mail. Between November 10 and December 9, 2011, 2428 public comments were made by 851 citizens. Of these, 687 supported the ordinance, 153 opposed it, and 11 neither supported nor opposed. A total of 202 people supported a 100% smoking ban, 144 opposed the “smoking separation”, and 23 opposed “smoking hours”. Comments were also submitted by JT, PM, and the Hyogo Medical Association (HMA). JT suggested that “DSRs provide adequate protection from SHS, and staff rooms are private spaces where smoking should be allowed”. PM agreed with the outline. HMA pointed out key differences between the committee’s proposals and the outline. For example, in the outline, the term “smoking ban” was replaced with “prevention of SHS” or the phrase “smoking separation is recommended” (Figure).^24^

Around 150 citizens mentioned that the draft ordinance represented a step backward from the committee report’s recommendations.^25^ On February 23, 2012, the draft ordinance prepared by the Governor was presented at the Hyogo Prefectural Assembly. Despite the criticism, the scope of the draft ordinance had remained unchanged (Figure).

The proposed ordinance was unanimously approved by 13 members of the Standing Committee of Health and Welfare on March 19, 2012, officially adopted by the Prefectural Assembly on March 21, 2012,^26^ and disseminated widely, using the media,^27^ internet, and promotional materials (eg, posters, leaflets, and stickers) (Figure).

### The ordinance

The ordinance encompasses 25 articles that, in order to prevent the negative health impact of SHS, established partial restrictions on smoking in public places.^12^ The level of restriction varies by type of establishment (Table 1). In type 1 and 2 establishments (eg, educational facilities for minors and health-care facilities), smoking is completely prohibited. Smoking is partially allowed for type 3 and 4 facilities, including tertiary educational institutions and hospitality premises over 100 m^2^. Smoking is permitted in type 5 establishments, such as hospitality facilities with an area less than 100 m^2^, as well as in an additional list of places that are considered exceptions (facilities regulated under the Act Regulating Adult Entertainment Businesses,^28^ such as Japanese pinball shops, night clubs, and adult entertainment shops, in addition to tobacco shops that include testing areas).

**Table 1.** Classification of targeted establishments under the Hyogo ordinance of 2012

Type	Areas where smoking must be prohibited	Conditions	Types of establishment
1	The whole area of the property, including outdoor premises	It is prohibited to use existing smoking rooms.	Educational establishments for persons under 18 years of age
	
2	The whole area inside buildings	Health-care facilities, government and municipal office buildings

3	Public spaces inside buildings	It is prohibited to establish new smoking rooms; however, an existing smoking room may be used **for the time being**.	Educational establishments for persons over 18 years of age, pharmacies

4	Public spaces inside buildings, as a general rule	A smoking area can be established in a part of the public space (less than two thirds of the public space) **for the time being**.	Public transport facilities, social welfare institutions, financial institutions, public meeting rooms, stadiums, athletic facilities, religious establishments, hotels, restaurants, hairdressing or beauty salons (>100 m^2^)

5		Smoking is permitted in the whole public space **for the time being**.	Hotels, restaurants, hairdressing or beauty salons (≤100 m^2^), theatres, cinemas, entertainment halls

Others	Facilities that fall under the Act Regulating Adult Entertainment Businesses are exceptions. This Act regulates the sex industry and amusement business for adults. Tobacco shops that include a testing area are also exceptions.		

The ordinance details the obligations of managers of targeted establishments.^12^ For instance, when managers find a person smoking in their establishment, they are required to ask the person to stop smoking or to leave. In addition, the Governor has the right to order on-site inspections. Penalties include a fine of up to 300 000 JPY (approximately 3000 USD) on owners or managers of establishments who fail to comply with the provisions, and up to 20 000 JPY (approximately 200 USD) on individual smokers.

The enforcement of the ordinance on types 1–3 facilities commenced on April 1, 2013, a year after the adoption of the ordinance, while a longer transition period was allowed for establishments of types 4–5, with a starting date of March 31, 2014. The fines on facilities of types 1–3 became effective on October 1, 2013, and fines for facilities of types 4–5 became effective on September 30, 2014. The expression *for the time being* is used in articles of the ordinance in reference to cases of smoking separation, without mention of a deadline.

The ordinance stipulates that Hyogo Prefecture is responsible for evaluating and potentially revising the ordinance 5 years after enforcement and for reviewing it every 3 years thereafter.^12^

## DISCUSSION

The ordinance, which only established a partial smoking ban, fails to provide effective protection against SHS and to cover the gap between the existing national policy related to SHS and WHO recommendations. A comparison between the Hyogo prefectural experience and the WHO recommendations was carried out to identify potential strengths and weaknesses of the legislation in Hyogo Prefecture.

The WHO’s recommendations were developed to provide local governments with guidance for becoming smoke-free; they include a model ordinance and “twelve steps” (Table 2, Table 3).^14^ The model ordinance draws on experience from many jurisdictions and from the implementation guidelines of the WHO FCTC Article 8. The model ordinance offers a set of comprehensive interventions in clear language for municipalities to use as a starting point towards smoke-free legislation (Table 3).^14^ The twelve steps summarize key actions necessary to make a city smoke-free through local legislation, based on lessons learned from case studies of subnational smoke-free initiatives around the world (Table 2).^14^ In 2012, 9 of the 100 most populous cities, including Melbourne (Australia) and Houston (United States), satisfied the WHO recommendation.^29^ East Asia is one of the world’s largest tobacco epidemic regions,^30^ but Beijing adopted an ordinance in 2014 completely banning smoking in all indoor public spaces.

**Table 2.** Comparison with the WHO model ordinance

	Key components of the model ordinance	Hyogo prefecture
1. Purpose	The ordinance protects the residents from the harmful effects of exposure to tobacco smoke in workplaces and public places.	(O) This ordinance mentions SHS in public places adequately, (x) but it does not mention SHS in workplaces yet.

2. Rationale	The Constitution guarantees the right to be healthy. International guidelines prompt to eliminate the source of smoke completely. (There is no safe level of exposure to tobacco smoke.)	(x) The ordinance does not mention the Constitution and international rules directly. (O) It mentions that SHS causes death and serious disease in nonsmokers.

3. Definitions	For the purposes of this ordinance, definitions such as public places, enclosed, smoke-free places etc need to be applied.	(O/X) A list of establishments is used rather than definitions of public enclosed places.

4. Prohibition of smoking in enclosed places	Smoking is prohibited in all enclosed public places and workplaces and within [a specified distance] of any entry, window or air intake of an enclosed public place or workplace.	(X) No distance to any entry, window or air intake of an enclosed public place. (The ordinance prohibits smoking in public spaces but not in the workplace. Workplace is covered by the Industrial Safety and Health Law in Japan.)

5. Prohibition in non-enclosed areas	Smoking is prohibited in non-enclosed, outdoor areas.	(O/X) The ordinance prohibits smoking outdoor areas of educational facilities only.

6. Duty of compliance	This sets out the specific actions and duties for which employers and businesses are responsible.	(O) This is covered.

7. Penalties and fines	Persons violating provisions of the ordinance are subject to fixed monetary penalties.	(O) This is covered.

8. Enforcement authority and inspections	The authority to enforce the provisions of the ordinance is defined. An inspector is also authorized.	(O) The governor has the right to order an officer to inspect. (X) The ordinance does not mention the enforcement authority.

9. Public complaints	The public shall be authorized to report violations of the ordinance to the inspection agency. They can call a telephone number to be displayed on signs and on the official web site.	(X) The ordinance does not mention public complaints.

10. Regulations	The governor may issue regulations for the effective implementation of the ordinance.	(O) The enforcement schedule is determined in detail by the supplement to the ordinance.

11. Reporting	The governor shall issue and publish an annual report on compliance with the ordinance.	(O) This is covered.

12. Entry into force	The ordinance states the day of publication and the day of enforcement.	(O) This is covered.

(o) Completely complies with the WHO’s model ordinance; (x) Does not comply with the WHO’s model ordinance; (o/x) Partially complies with the WHO’s model ordinance.

**Table 3.** Comparison with the WHO “Twelve steps”

Twelve steps	Hyogo prefecture
1. Set up a planning and implementation committee	(O) Hyogo Prefecture’s DoHW established a special committee in June 2010: Hyogo Prefecture Consultation Committee for Second-hand Smoke Prevention Measures.

2. Become an expert	(O) Hyogo established an “SHS Prevention Office” in the Department of Health Promotion to research smoke-free interventions.

3. Involve local legislative experts	(O/X) The committee did not include a person from the law field. The officers responsible for legislation attended the meetings. The legal department only checked the draft.

4. Study several potential legal scenarios	(X) Legal action by the tobacco industry was not considered.

5. Recruit political champions	(X) The Governor of Hyogo worked to promote the ordinance, but we did not find evidence of any promotion in the media record (the newspapers and magazines).

6. Invite the participation of civil society organizations	(O) A non-profit organization for children in Hyogo and the Hyogo Women’s Association were invited to the committee. The chairperson of the committee is also the chairperson of the Tobacco Control Medical-Dental Research Network.

7. Work with evaluation and monitoring experts	(O/X) The DoHW did not work with evaluation or monitoring experts, but national survey data (Comprehensive Survey of Living Conditions) are available.

8. Engage with media and communications experts	(O) The prefecture is working aggressively with the media, and the officers of the SHS Prevention Office are promoting the prevention of SHS.

9. Work closely with enforcement authorities	(O/X) The Governor has the right to order an officer to inspect a potential violation, but the prefecture did not work with enforcement authorities, such as the police or special inspectors, and did not design a clear protocol for inspections. The ordinance only established penalties and fines.

10. Develop and disseminate guidelines	(O) The prefecture announced the implementation date for legislation by means of the media, a website, and promotion materials (eg, posters, leaflets, and stickers). It also held an explanatory meeting for owners and managers of hospitality businesses. Furthermore, a contest for creating a slogan was held.

11. Celebrate the implementation day	(X) Hyogo has not celebrated the implementation day yet.

12. Ensure maintenance of the law	(O/X) The ordinance specifies that it is mandatory to maintain the ordinance for at least 5 years after it comes into force, with reviews every 3 years thereafter. However, monitoring of compliance, public opinion, indoor air quality, and workers’ health and economic impact following implementation of the ordinance is not specified.

DoHW, Department of Health and Welfare.

(o) Completely complies with the WHO’s step; (x) Does not comply with the WHO’s step; (o/x) Partially complies with the WHO’s step.

Hyogo’s draft ordinance was modified considerably from the committee recommendations. The most striking difference is the adoption of a partial restriction rather than a complete ban as recommended. Allowing smoking separation *for the time being* could hinder future efforts to move towards a complete smoking ban. Duties to post a sign indicating that smoking is permitted in smoking areas were added in the draft ordinance. Another difference was an increase in the number of facilities exempted from the regulation. The threshold for the restaurants to be covered by the regulation was increased from 75 to 100 m^2^. Smoking restriction was only to be applied to those places with areas over 100 m^2^, the same regulatory level as the Kanagawa ordinance, which allows 80% of restaurants in Hyogo Prefecture to be exempted.^31^

A comparison of WHO’s model ordinance with the Hyogo ordinance reveals additional gaps (Table 2). For instance, the WHO model recommends explicit prohibition of smoking in workplaces and in non-enclosed or outdoor areas adjacent to smoke-free areas.^14^ Workplaces are covered by the Industrial Safety and Health Law (ISHL) in Japan. Discussion of prohibition of smoking in workplaces was avoided, since they are beyond the jurisdiction of the health department.^32^ However, the amended ISHL in 2014 promoted *smoking separation* officially.^33^ Another of the omitted recommendations is a system that allows the public to report violations or suspected violations for inspection.

Nevertheless, several recommendations are followed, at least partially. For instance, the obligation of compliance is clear, as are penalties, regulations, and reporting; implementation guidelines were established with the ordinance; and the Hyogo ordinance does mention the harmful effects of exposure to tobacco smoke. This is an important change in comparison with ordinances on street smoking bans, which had been common measures taken by local governments and which stressed littering and manners rather than health concerns.^6^ However, the ordinance states that “the right of smokers” was also taken into account, thus weakening the public health argument.

Analysis of the policy development process provides insight into the passing of a regulation not compliant with WHO FCTC. The process followed in Hyogo Prefecture was compared with the corresponding WHO recommended 12 steps (Table 3), most of which were not fully followed in Hyogo Prefecture.

Setting up the consultative committee partially covered two of the 12 steps: establishing a planning and implementation committee and inviting the participation of civil society organizations. However, both the mandate and the membership of the committee made it difficult to enact a 100% smoking ban. The mandate of the committee was restricted to drafting a suggestion for the ordinance and did not include planning and implementing the regulation, except for issues covered in the legislation itself.

The SHS Prevention Office was in charge of studying smoke-free interventions in other jurisdictions, thereby covering the recommendation on learning experiences of others. This office vigorously promoted the prevention of SHS through the media, in accordance with the recommendation to develop and disseminate messages that promote the legislation to the public. Signs and leaflets were prepared to announce and facilitate the implementation of the ordinance, and Hyogo Prefecture held an explanatory meeting for owners and managers of hospitality businesses, as recommended in the tenth step.

Another recommendation advises ensuring maintenance of the law. The Hyogo ordinance specifies that periodic mandatory review of the text of the ordinance is required. However, monitoring of compliance, public opinion, indoor air quality, and economic impact were not specifically addressed in the ordinance or the implementation guidelines. We found that a national survey, the Comprehensive Survey of Living Conditions, is conducted every 3 years, and its results are available for examination.^34^ No evidence was collected to prove that Hyogo Prefecture followed other recommendations, such as steps 4, 5, and 11 (Table 3).

In the committee, the hospitality sector representatives, who opposed regulation from the beginning of the process, were an obvious barrier to drafting and implementing effective regulation. Moreover, the opportunities provided to representatives of the tobacco industry to present their views made the committee into a forum seeking consensus between those arguing for evidence-based tobacco control and those opposing regulations on the basis of perceived economic risks. This internal contradiction limited the capabilities of the committee to propose an evidence-based regulation following the WHO FCTC. The health effects of SHS exposure have been established; the U.S. Environmental Protection Agency, the U.S. National Toxicology Program, the U.S. Surgeon General, and the International Agency for Research on Cancer have all classified SHS as a known cancer-causing agent.^35^

As expected, JT opposed the use of legislative measures to prevent exposure to SHS in public spaces, arguing that “current evidence cannot prove that SHS has adverse health impacts”, advocating for “the coexistence of smokers with nonsmokers” through separate smoking areas, and emphasizing concerns about negative economic impact.^21^ These actions indicate that tobacco industry interference exists at the local level and uses strategies similar to those reported in a national context in other countries.^10^^,^^36^^,^^37^

Several committee members involved in health professional associations voiced their concerns and provided evidence in support of smoking bans. Additionally, a representative of a tobacco control non-governmental organization participated in the committee, and public forums were organized by the HMA and HTFA (in June 2011 and June 2012 for World No Tobacco Day). No other actions advocating for stronger legislation or to counteract industry tactics were noted. Although representatives of civil society participated in the committee, they remained shy advocates for the ordinance.

In contrast, in many other jurisdictions, civil society has played an important role at the local level in advocating for tobacco control. For instance, in Almaty, Kazakhstan, the National Coalition for “Smoke-Free Kazakhstan” lobbied for a budget for the city’s smoke-free program and played a key role in developing legislation and providing support for the approved program.^38^ In Chandigarh, India, the Burning Brain Society filed petitions against city government offices for violating the existing national smoke-free provisions and urged the city government to begin full enforcement.^39^

Another interesting factor identified was the role of the national government. Hyogo Prefecture was partially encouraged by the letter from the MHLW in 2010,^19^ which stressed the importance of a 100% smoking ban to prevent SHS exposure. Nevertheless, by allowing *smoking separation* as a valid measure and offering implementation funds, it is likely that the letter diminished the potential for 100% smoking bans. The Ministry of Finance being the major shareholder of JT might be a hindrance to stronger tobacco control at the national level.

Following the initiatives in Kanagawa and Hyogo Prefectures, Osaka Prefecture in 2013 and the Tokyo Metropolitan Area in 2014 experienced similar stalls and opposition to making local smoke-free ordinances.

### Conclusion

This case study revealed that Japan faces several challenges limiting the enforcement of 100% smoke-free environments. First, the national government provides weak guidance to prefectures and municipalities by allowing certain types of establishment to introduce nonprotective *smoking separation*, thus hindering the potential to be 100% smoke-free. Second, the involvement of civil society in the development and adoption of a law was very limited in comparison with other international experiences, where such organizations were closely involved in smoke-free interventions. Third, there is strong interference by the tobacco industry, both directly and indirectly through the hospitality sector, which hinders efforts to adopt effective policy.

## ONLINE ONLY MATERIAL

Abstract in Japanese.
