# Supplementary material for: Industry Speed Bumps on Local Tobacco Control in Japan? The Case of Hyogo
Source: J Epidemiol. 2015 Jul 5;25(7):496–504. doi: 10.2188/jea.JE20150001 (PMC4483376; doi:10.2188/jea.JE20150001)
Supplement: Abstract in Japanese. [file je-25-496-s001.pdf]

日本における地方自治体主導のたばこ規制　～兵庫県の事例

山田　恵子[1,2,3]、森　渚[1,4]、柏原　美那[5]、安田　咲こ[6]、堀江　瑠美[7]、大和　浩[8]、ロイク・ギャルソン[1]、フランシスコ・アルマダ[1]
１　世界保健機関（WHO）構健康開発総合研究センター（WHO 神戸センター）
２　大阪大学大学院　医学系研究科　社会医学講座　公衆衛生学
３　兵庫県　健康福祉部　健康局　医務課
４　東京大学大学院　医学系研究科　社会予防疫学分野
５　世界保健機関（WHO）西太平洋地域事務局
６　京都大学 大学院医学研究科　社会健康医学系専攻　国際保健学講座　社会疫学分野
７　世界銀行東京開発ラーニングセンター
８　産業医科大学　産業生態科学研究所 健康開発科学研究室

抄録
背景　日本は「たばこの規制に関する世界保健機関枠組条約」を 2004 年に批准したが、その第 8 条である公共の場所における「たばこの煙にさらされることからの保護」を未だ完全に実施できていない。そればかりか、日本政府は公共の場所における喫煙室の設置を妥当な方策であると許容している。さらに、地方自治体によるたばこ規制も日本においては限定的である。本研究は 2012 年の兵庫県によるたばこ規制の取り組み（受動喫煙防止条例の制定）を分析し、公共の場における禁煙環境の達成において何が障壁となっているのかを明らかにした。
方法　記述的ケーススタディの手法を用い、受動喫煙防止条例の成立過程を分析した。データは議事録や、公的記録、並びに施策関係者に対するインタビューを用いた。
結果　兵庫県は受動喫煙防止の条例化を提議するための委員会を設立し、ほとんどの委員会メンバーは飲食店等を含む公共的な場所（屋内）の完全な禁煙化に賛成した。しかし、委員のうち飲食業と宿泊業の代表者は条例による禁煙に反対した。加えて、委員会会議でたばこ会社に発言の機会が設けられた。さらに、条例化の最中に開催された地方選挙によって県議会の政権が交代し、このことも公共的な場所の完全な禁煙を定めた条例の制定に対して影響した。また、条例制定の過程を通して、市民団体による公共的な場所（屋内）の完全禁煙化を求める働きかけも他国と比して不活発であった。最終的に県議会が制定した条例は、委員会の提案内容より規制が弱い、広範囲の例外規定を含んだものとなった。
結論　兵庫県の取り組みを分析することで、三つの要素（不完全な国の法規制、市民団体による活動の弱さ、たばこ産業による干渉が明らかになった。これらは他国における今後の教訓となり、我が国のたばこ規制における重要な課題ともいえる。

キーワード　喫煙。タバコの煙による汚染。法規制。地方自治体。日本。
